# Titanium-Pillared Clay: Preparation Optimization, Characterization, and Artificial Neural Network Modeling

**DOI:** 10.3390/ma15134502

**Published:** 2022-06-26

**Authors:** Seyed Heydar Mosavi Mirak, Seyedmehdi Sharifian, Fatemeh Esmaeili Khalil Saraei, Neda Asasian-Kolur, Bahram Haddadi, Christian Jordan, Michael Harasek

**Affiliations:** 1 Fouman Faculty of Engineering, College of Engineering, University of Tehran, Fouman 43516-66456, Iran; heydarmosavy@gmail.com (S.H.M.M.); s.m.sharifian@gmail.com (S.S.); f.esmaeili.kh@ut.ac.ir (F.E.K.S.); 2 Institute of Chemical, Environmental and Bioscience Engineering, Technische Universität Wien, Getreidemarkt 9/166, A-1060 Vienna, Austria; bahram.haddadi.sisakht@tuwien.ac.at (B.H.); christian.jordan@tuwien.ac.at (C.J.); michael.harasek@tuwien.ac.at (M.H.)

**Keywords:** pillared clay, intercalation, titanium, characterization, artificial neural network

## Abstract

Titanium-pillared clay (Ti-PILC), as one of the most suitable types of porous adsorbents/(photo)catalysts, was prepared from a local type of Iranian clay and titanium isopropoxide. The production process was optimized by changing three operating parameters, including the clay suspension concentration (in the range of 0.5–10% *w*/*v*), the H^+^/Ti ratio (2–8 mol/mol), and the calcination temperature (300–700 °C). The largest specific surface area for the Ti-PILC was about 164 m^2^/g under the clay suspension of 0.5% *w*/*v*, H^+^/Ti = 6, with a surface area 273% larger than that of the raw clay. The surface areas obtained from more concentrated clay suspensions were, however, comparable (159 m^2^/g for 3% *w*/*v* clay and H^+^/Ti = 4). An increase in the calcination temperature has a negative effect on the porous texture of Ti-PILC, but based on modeling with artificial neural networks, its contribution was only 7%. Clay suspension and H^+^/Ti ratio play a role of 56 and 37% of the specific surface area. The presence of rutile phase, and in some cases anatase phase of TiO_2_ crystals was detected. FTIR and SEM investigations of Ti-PILCs produced under different operating parameters were analyzed.

## 1. Introduction

Clay, as an abundant natural material, can be used in various adsorption and catalytic applications [1]. Bentonite is a clay type from the smectite subgroup mainly containing montmorillonite. Due to its relatively high porosity, large specific surface area, and significant thermal resistance, bentonite is widely used in industrial processes [2]. However, it has main limitations in commercial uses, including impermanent and unstable structure and porosity, especially in high-temperature applications. Therefore, some methods are recommended to improve the properties of clays and make them more reliable for large-scale applications. Pillarization is a suitable technique for this purpose and is based on establishing stable ions/molecules within the bentonite layers [3,4]. The pillaring process is strongly influenced by the type of pillaring agent and the operating conditions. Generally, it increases the basal spacing of clays, the surface area, and the pore volume [5].

Pillared clays (PILCs) have attracted much attention for years due to their unique catalytic properties. However, their use as adsorbents for environmental targets has also attracted increased attention recently. The removal of heavy metals such as arsenic [6], lead (II) [7], zinc, and chromium [8]; organic and inorganic pollutants such as phenol complexes [9] and carbon monoxide [10]; and pharmaceutical contaminants such as amoxicillin and imipramine [11] are examples of the use of PILCs as adsorbents. The catalytic ability of PILCs to improve the oxidation reaction of NO [12], phenol [13], paracetamol [14], toluene [15], and CO_2_ [16] is documented in previous studies.

Besides the oxide sols, poly(oxycations) are the essential pillaring agents for producing highly heat-resistant PILCs. The literature review confirmed that titanium [17], iron [18], zirconium [19], and aluminum poly(oxycations) [20] are some types of pillaring agents that can be used for PILC synthesis. Ti-PILC results from cation exchange between clay and titanium poly(oxycation) and is formed by hydrolysis of titanium compounds followed by ion exchange and calcination processes [21]. The literature review showed that Ti-PILC, modified by KI, has recently been used for Hg elimination from gas mixtures [22]. In addition to the gas phase applications, the use of Ti-PILC in the oxidation of methylene blue by a synthetic effluent has been reported [23]. They modified Ti-PILC by doping Ag on the adsorbent surface to improve system performance.

Different titanium sources, including titanium chloride, ethoxide, and isopropoxide, were used for Ti-PILC production. Since the properties of pillared clay are strongly influenced by the operating parameters, some studies consider the search for optimal synthesis conditions. Bernier et al. proposed a process for synthesizing montmorillonite-pillared clay with TiCl_4_. They optimized effective parameters such as pH, calcination temperature, and titanium concentration in suspension. The best result was achieved using a 4% clay suspension accompanied by an HCl and TiCl_4_ solution to achieve a ratio of 10 mmol Ti/g montmorillonite at room temperature while mixing the solution for 3 h. The calcination at 600 °C led to the collapse of the zeolitic structure of clay and a large surface area with even pillar distribution [24]. Process optimization of Ti-PILC synthesis from bentonite using titanium ethoxide is also reported. For this purpose, the effects of several parameters, including the weight percentage of the clay suspension (0.1–0.2%), the temperature of the pillaring solution production (10–65 °C), HCl/Ti molar ratio (1.5–3), Ti/clay ratio (5–25 mmol/g), calcination temperature (60–500 °C), and the rate of addition of pillaring solution to clay solution (0.4–1.3 cm^3^/min) were investigated. The PILC prepared at a temperature of 25 °C, HCl/Ti = 2.5, Ti/Clay = 15 mmol/g, and a clay suspension of 0.15% showed a relatively large surface area and micropore distribution [25]. It has been shown that using TiCl_4_ leads to PILC with a large surface area (323 m^2^/g) and a microporous texture. In contrast, titanium isopropoxide produces mesoporous PILC with larger pore volumes [26].

Del Castillo et al. compared the effect of different titanium alkoxides and hydrolyzing acids on the performance and properties of the Ti-pillared montmorillonite. Titanium sources showed an essential role in basal distances and specific surface area of the final products. Additionally, the acid/alkoxide mole ratio was one of the critical factors. Titanium (IV) ethoxide led to the best textural and thermal stability properties due to its most accessible interchange compared to other alkoxy groups (such as propoxide and butoxide). They found that the best calcination temperature to achieve the highest specific surface area was 100 °C, giving a surface area equal to the summation of S_BET_s of TiO_2_ and clay S_BET_s. However, this temperature seems insufficient for stabilizing the structure of pillared clay. The sequence obtained for the specific surface areas obtained for different titanium sources is as follows: titanium ethoxide > titanium normal propoxide > titanium isopropoxide > Ti-normal butoxide. The S_BET_ of the Ti-isopropoxide-pillared montmorillonite was between 146 and 273 m^2^/g at calcination temperatures between 700 °C and 100 °C [27]. Yoda et al. used a two-step method for the preparation of titanium-pillared clay as a catalytic adsorbent for toxic VOCs in air, including preparing a hydrophobic internal structure for the clay and then intercalation of titanium isopropoxide dissolved in supercritical CO_2_, followed by hydrolysis with the adsorbed water present in the interlayer space. Hydrophobization of the interlayer space had an essential role in the intercalation results. Alkyltetramethylammonium ions were selected for the control of interlayer space. The controllable titanium isopropoxide hydrolysis in the interlayer space of clay led to a more dispersed introduction of the titanium oxide particles. Depending on the number of carbons in alkyl chains, the specific surface area of the Ti-pillared clay was between 60–212 m^2^/g [28]. The advantages of using supercritical CO_2_ instead of conventional solvents have been previously investigated, revealing: easier and smoother transfer of solute through the interface, low surface tension and viscosity, controlling solvent properties with changing temperature and pressure, and no competitive adsorption of solvent on the porous material [29].

Manova et al. prepared new nonstructural titania-clay porous materials based on different clay materials such as montmorillonite, iron-rich smectite, and vermiculite. They used titanium isopropoxide as the titanium source to reach porous clays in the range of 66–105 m^2^/g. The use of tetramethoxysilane as a silica precursor and titania precursor was suggested to prepareTiO_2_/SiO_2_-clay materials with higher specific surface areas in the range of 290–300 m^2^/g. The photocatalytic activity of these materials for 2,4-dichlorophenol decomposition in an aqueous phase solution was studied and was found superior to that of commercial TiO_2_ P-25 anatase [30]. Zhou et al. used sepiolite clay as raw material to produce Ti-PILCs. It is reported that Ti-PILCs produced under the condition Ti/clay = 40 mmol/g showed a significantly better photocatalytic performance than other ratios [31]. The same study was performed to optimize the Ti/clay ratio for the Ti-PILC synthesis process to remove Cr from wastewater [3]. Pichat et al. used the Ti-montmorillonite prepared by titanium tetraisopropoxide as a photocatalyst for aqueous phase photodegradation of 4-chlorophenol and gas phase removal of methanol in air. They found that the Ti-pillared clay had higher efficiency for methanol removal in air compared to commercial TiO_2_ P-25; however, its ability in the aqueous phase was decreased. The highest performance was obtained at a Ti/clay ratio of 10 mmol/g and calcination of Ti-intercalated clay by microwaves [32].

In this work, titanium isopropoxide and hydrochloric acid are considered pillaring agents. Few studies worked on determining the effects of parameters and optimization of the conditions in this system. A significant research gap is related to the selection of very dilute clay suspensions in previous studies, as the use of concentrated clay suspensions is in high demand on a large production scale. In the current study, relatively wide ranges of levels of effective parameters are selected and investigated. The effects of parameters, including the clay suspension concentration, acid/Ti molar ratio, and calcination temperature, on the properties of Ti-PILC, were evaluated by selecting four levels for each factor and performing a total of 64 experiments to obtain a comprehensive overview of the Ti-PILCs synthesis system.

In addition to experimental analysis, applying a mathematical approach is also valuable for determining the relationships between the parameters and factors that play a role in an empirical system. An artificial neural network (ANN) is a computational model based on the simulation of biological systems that can be used to evaluate and model empirical data [33]. This tool can predict the performance of both linear and nonlinear systems. ANN prediction of system behavior is accurate when a sufficient number of experimental data are available [34,35]. A model is proposed to analyze the system using the experimental optimization data obtained in the present work and ANN.

## 2. Materials and Methods

The raw bentonite used in this research was supplied by Dr. Mojallali Industrial Chemical Complex Co., and the other materials were supplied by MERCK Co. (Darmstadt, Germany). The raw clay’s cation exchange capacity (CEC) was obtained at approximately 92 meq/100 g clay. An XRF analysis was performed to determine the elemental content of the raw clay, and the contents of Si and Al were about 66.5 and 11.0%, respectively, and Ca, Fe, Na, Mg, K, Ti, Mn, and P were also found in the clay.

### 2.1. Preparation of Pillared Clay

**Figure 1:**
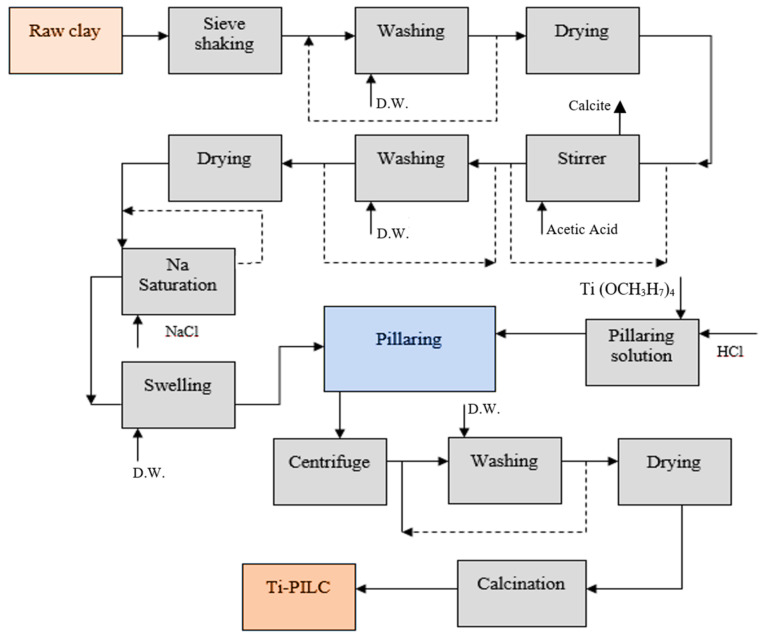
Schematic of titanium-pillared clay production process.

The preparation of pillared clay through a stepwise procedure is shown in Figure 1 and explained below. The raw bentonite is physically sieved through a stainless-steel screening machine to obtain an average particle size of less than 45 μm. Then, the primary washing of the raw clay is carried out 3 times with a suspension ratio of 1/5 (*w*/*v*) for clay/distilled water for 3 h by stirring at ambient temperature. The washed clay is dried in an oven at 110 °C for 12 h. The clay is then stirred at ambient temperature for 30 min in the presence of acetic acid (0.2 M) to remove calcite from the clay structure. This step is repeated twice until no bubbles are observed when acid is added. The samples are then washed three more times with distilled water to separate the residual acid from the samples. They are then dried in an oven at 110 °C for 12 h.

In order to achieve a perfect exchange between the metal cations available in the structure of the bentonite clay with sodium, the purified clay from the previous section is stirred in the presence of NaCl 1 M at room temperature for 12 h. For chlorine removal from the exchanged bentonite, it is washed three times with distilled water. The Na saturation procedure is repeated twice, and then the samples are dried in the oven at 110 °C for 12 h.

The method used in the present work for clay pillarization by Ti-containing solution is inspired by the method already used by Bineesh et al. with some modifications [36]. The Na-saturated clay suspension is prepared in four different weight percentages (0.5, 1, 3, and 10 g clay/g water) to investigate the effects of the concentration of suspension on the pillaring process. The suspensions are kept for 12 h under room conditions to allow the clay to swell accordingly. In addition, the pillaring solution is synthesized by titanium propoxide and a constant Ti/clay ratio of 10 mmol/g. For each of these prepared clay suspensions, a certain amount of the pillaring solution is added dropwise to the HCl 2 M solution using a stirrer until different H^+^/Ti ratio values (2, 4, 6, and 8) are reached. The pillaring solution is kept under ambient conditions for 15 h under aging. It is then added dropwise to the clay suspension and stirred for 24 h until the ion exchange process is complete. A centrifuge is used to separate the intercalated clay from the solution. Washing with distilled water is carried out three times to remove the chloride from the solid phase. The dried end product is fed into a tube furnace for calcination. The temperature range for calcination is from 300 to 600 °C (4 levels with intervals of 100 °C) for 3 h with a heating rate of 3 °C/min.

### 2.2. Characterization Methods

The surface characterization of synthesized Ti-PILCs was performed with different analytical instruments. The porous properties were determined by N_2_ adsorption/desorption tests with an automated volumetric device (BELSORB MINI II). Before the adsorption/desorption tests, the degassing process was carried out at 200 °C for 4 h on the predried synthesized Ti-PILC samples. The BET specific surface area, the total pore volume, and the mean pore diameter of the pillared clays produced under different operating conditions were calculated and compared. Furthermore, the micro- and mesopore volumes and the pore size distributions were determined for each sample using the HK, BJH, and DFT methods.

In addition to measuring the specific surface area and other porous properties for all 64 samples, some samples were selected for further analysis. Because of the results of the sensitivity analysis tests, which indicated a relatively small role of the calcination temperature compared to the two other parameters, eight samples were selected to investigate how the change of the two main factors caused this considerable influence. It means that two separate groups of samples were selected: firstly, samples with a calcination temperature of 300 °C and a clay suspension of 0.5% (*w*/*v*) with different H^+^/Ti ratios (2, 4, 6, and 8 to produce Ti-PILCs called 0.5-2-300, 0.5-4-300, 0.5-6-300, 0.5-8-300), and secondly, a group of samples with a minimum calcination temperature of 300 °C and an H^+^/Ti ratio of 3 with different percentages of clay suspension (0.5, 1, 3, and 10 to produce Ti-PILCs including 0.5-4-300, 1-4-300, 3-4-300, 10-4-300).

The above samples were subjected to XRD, XRD, FTIR, and FESEM analyses. The XRF analysis was performed with a Philips PW1730 spectrophotometer to determine the element content of Ti-PILCs. XRD was also used to study their crystalline/amorphous phase and the role of pillaring agents to increase the interlayer distances. In addition, the functional chemical groups of the synthesized Ti-PILCs were analyzed with the FTIR spectrophotometer (Perkin Elmer, model: SPRCTROM). Finally, the porous texture, particle size, morphology of the nominated samples, and the percentage of surface elements were determined with the FESEM (TESCAN, model: MIRA III).

## 3. Artificial Neural Network Modeling

**Figure 2:**
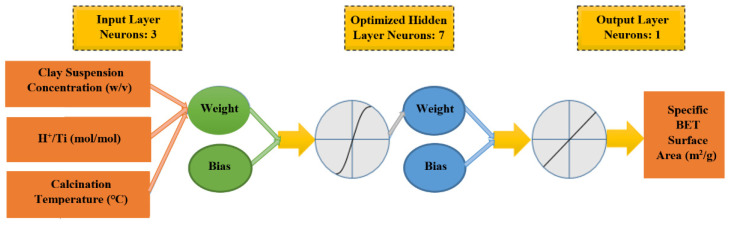
The structure of ANN model designed in the present work.

In this study, a 3-layer feedforward ANN is used by a backpropagation algorithm with a tangent sigmoid transfer function (tansig) on the hidden layer and a linear transfer function (purelin) on the output layer. The 64 test points are categorized into three groups: 41 points for training, 13 for validation, and 10 for the test. As explained in the experimental section, the range of change of the clay suspension concentration, H^+^/Ti ratio, and calcination temperature as members of the input layer is 0.5–10% (*w*/*v*), 2–8 (mol/mol), and 300–600 °C, respectively. Under these conditions, the analyzed specific surface area as an element of the output layer changes between 57.6 and 163.6 m^2^/g. The structure of the designed ANN with seven neurons in the hidden layer is shown in Figure 2.

All values (X_i_) were normalized with the following equation in the range of 0.1–0.9:

(1) $$N_{i}=\frac{0.8\left(X_{i}-X_{min}\right)}{X_{max}-X_{min}}+0.1,$$

where *N_i_*, *X_max_*, and *X_min_* are the normalized value, the maximum value, and the minimum value corresponding to each instance of experimental data (*X_i_*), respectively. The parameters indicating the model performance are the correlation coefficient (*R*) and the mean square error (*MSE*), which are defined by the following equations:

(2) $$R=\sqrt{1-\frac{\sum_{i=1}^{N}{\left(X_{i}-X_{i,p}\right)}^{2}}{\sum_{i=1}^{N}{\left(X_{i}-{\bar{X}}_{i}\right)}^{2}}},$$

(3) $$MSE=\frac{1}{N}\sum_{i=1}^{N}{\left(X_{i,p}-X_{i}\right)}^{2},$$

where *X_i_* is the experimentally measured value, *X_i,p_* is the value predicted by the model, ${\bar{X}}_{i}\;$is the mean value, and *N* is the number of test points.

**Figure 3:**
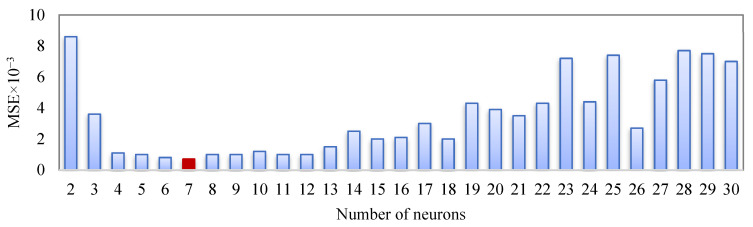
The optimum number of neurons in the hidden layer.

The performance of the ANN model is highly dependent on the number of hidden neurons. For optimization of the number of neurons in the hidden layer, the relationship between the number of neurons in the hidden layer and MSE is shown in Figure 3. It can be seen that the Levenberg–Marquardt algorithm (LMA) with seven hidden layer neurons shows, relatively, the best performance in the ANN architecture.

## 4. Results and Discussion

### 4.1. Effects of Operating Parameters and Optimization

**Figure 4:**
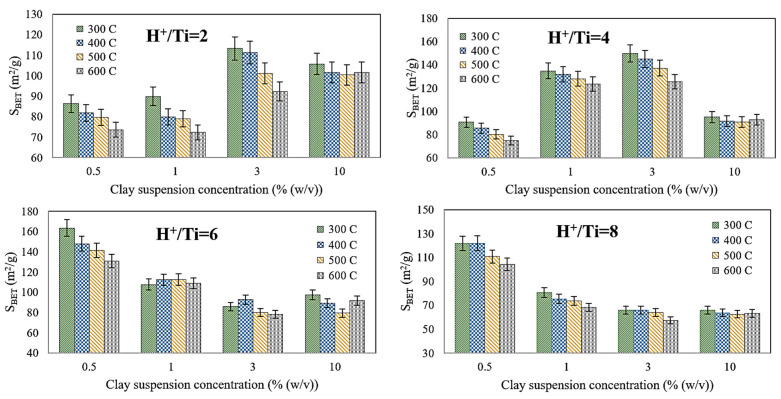
Effects of clay suspension concentration on the specific surface area of Ti-PILC at different ratios of H^+^/Ti and calcination temperatures.

As mentioned in the previous section, three operating parameters expected to influence the Ti-PILC synthesis process significantly were selected. Each parameter changed at four levels, and 64 BET tests were performed. The results are shown in Figure 4.

Figure 4 shows the changes in specific surface area at different concentrations of the clay suspension, H^+^/Ti ratios, and calcination temperatures. It was observed that the calcination temperature is not a relatively influential factor, especially when the H^+^/Ti ratio and the percentage of clay suspension are high (8 mol/mol and 10% (*w*/*v*), respectively). In general, the temperature influence on the specific surface area of Ti-PILC decreases with increasing H^+^/Ti ratio. A lower calcination temperature results in an adsorbent with a relatively higher specific surface area, especially when diluted clay suspension is used. In contrast, a high calcination temperature (higher than 300 °C) leads to lower porous properties of the adsorbent, additional energy consumption, and higher operating costs, which are not economically beneficial. A literature review shows that the crystallite size of TiO_2_ particles also increases when the calcination temperature increases from 300 to 600 °C [37]. This phenomenon consequently increases the volume of large mesopores of PILC and reduces the specific surface area. Basoglu showed that the adverse effects of calcination temperature between 200 and 500 °C under low H^+^/Ti (2.5) and high H^+^/Ti ratio (4.0) were about 15 and 7% [26]. More different behaviors were obtained under different operating conditions in the present work. Depending on the H^+^/Ti and clay suspension used, the calcination temperature strongly or mildly negatively influenced the S_BET_ and was ineffective in some cases.

At low H^+^/Ti ratios, the highest S_BET_ results were obtained at medium clay suspension percentage (3% (*w*/*v*)), while at high H^+^/Ti ratios, the maximum S_BET_ results were obtained at minimal clay suspension percentage (0.5% (*w*/*v*)). Furthermore, the trend of the low H^+^/Ti ratio curves (2 and 4) are significantly different from those when H^+^/Ti ratios are maintained at high levels (6 and 8). At low H^+^/Ti ratios (2 and 4), by increasing the concentration of the clay suspension, the specific surface area of Ti-PILC increased up to its maximum value was 3% (*w*/*v*). After this point, the slope of the curve changes from positive to negative. On the contrary, at high H^+^/Ti ratios, the maximum BET surface areas can be obtained at a very low clay suspension ratio (0.5% (*w*/*v*)), and by increasing the clay suspension concentration, a sudden drop in S_BET_ results was observed (from 0.5 to 1% (*w*/*v*)). In addition, a further increase in the clay suspension concentration had a slight effect on the specific surface area of the product.

Figure 4 shows that the H^+^/Ti ratio is a relatively more effective parameter at low clay suspension concentrations. The best performance of the pillaring process and the highest surface area was achieved for Ti-PILC synthesized at a clay suspension concentration of 0.5% (*w*/*v*) and an H^+^/Ti ratio of 6, while for more concentrated clay suspensions, the corresponding values were obtained at an H^+^/Ti ratio of 4 and 2, respectively. The role of H^+^ or acid in synthesizing Ti-PILC is titanium isopropoxide hydrolysis. Diluted and concentrated clay suspensions are prepared by dissolving specific amounts of clay in the required water. For the constant value of Ti/clay used in all experiments (10 mmol/g), the amount of titanium isopropoxide available for hydrolysis is the same. Therefore, to achieve the pH of the solution up to a certain amount necessary for hydrolysis of titanium isopropoxide in diluted clay suspensions, a higher acid volume (H^+^/Ti) is required. The data show that if diluted clay suspensions are used, an H^+^/Ti ratio of 6 is necessary, as it refers to the complete hydrolysis process. In contrast, concentrated clay suspensions require a smaller amount of acid or a lower H^+^/Ti ratio.

The experimental results showed that the highest specific BET surface area was achieved with a 0.5% (*w*/*v*) clay suspension, a ratio of 6 for H^+^/Ti, and a calcination temperature of 300 °C. This Ti-PILC sample has a specific surface area of 160 m^2^/g. Other porous properties such as total pore volume, N_2_ adsorption/desorption isotherms, and pore size distributions are discussed in Section 4.3. The data also showed that achieving Ti-PILC with a competitive specific surface area of about 150 m^2^/g is possible, with a higher clay suspension concentration (3% (*w*/*v*)), an H^+^/Ti ratio of 4, and a calcination temperature of 300 °C.

A literature review was conducted to compare the performance of the pillarization procedure used in the present study with those reported previously; the results are presented in Table 1. Various types of titanium sources were used to produce Ti-PILC, including titanium tetrachloride (TC), titanium (tetra)isopropoxide (TIP), titanium butoxide (TB), and titanium ethoxide (TE). The raw clays used in these studies were exclusively bentonite and/or montmorillonite. Kitayama et al. reported the largest surface area of the samples modified with titanium isopropoxide, which was approximately 404 m^2^/g. However, due to the relatively high porosity of the raw bentonite (saponite) in this case (152 m^2^/g), the increasing proportion of S_BET_ after pillaring was only 166% [38]. The pillaring method presented in the present paper increased the specific surface area to about 273%.

### 4.2. Relative Contribution of Operating Parameters

**Figure 5:**
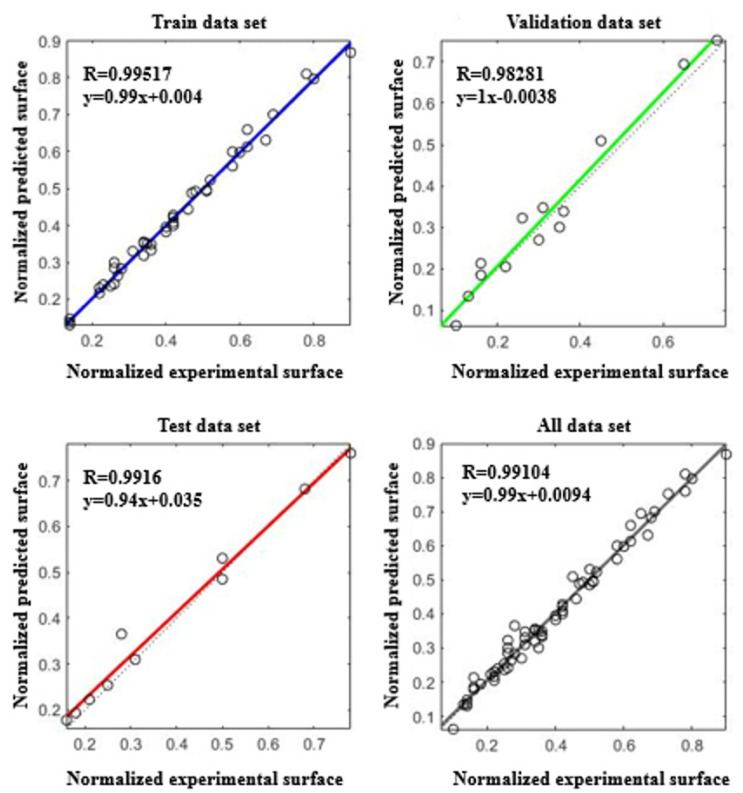
Comparison of the normalized experimental and ANN predicted data for different data sets: training, validation, test, and all data sets.

**Figure 6:**
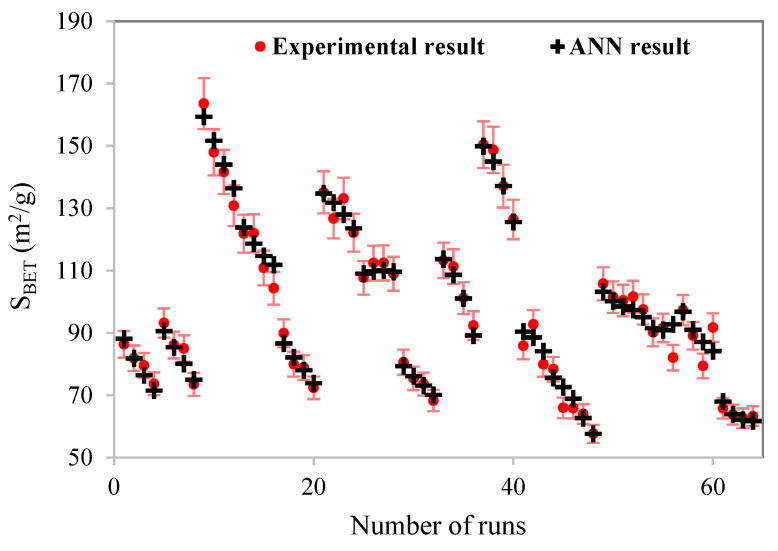
Comparison of all experimental results and ANN model predicted values.

Figure 5 illustrates the ANN-predicted normalized specific surface area versus the corresponding normalized experimental data for different data sets: training, validation, test, and all data sets. Validation, test, and overall data curves with R^2^ larger than 0.98 show the satisfying performance of the ANN model. Figure 6 is further evidence of the excellent agreement of the ANN model with all 64 experimental data. One of the most expected results of the ANN model is the determination of the quantitative effectiveness and the contribution of the individual operating parameters to system performance. These values are calculated using the Carson formula as follows [46,47]:

(4) $$Q_{ik}=\frac{\sum_{j=1}^{L}\left(\frac{w_{ij}}{\sum_{r=1}^{N}w_{rj}}\;\;v_{jk}\right)}{\sum_{i=1}^{N}\left(\sum_{j=1}^{L}(\frac{w_{ij}}{\sum_{r=1}^{N}w_{rj}}\;\;v_{jk}\right))},$$

where *w_ij_* is the connecting weight between the input neuron *i* and the hidden neuron *j*, and *v*_jk_ is the connection weight between the *j*’th hidden unit and the *k*’th unit. Additionally, $\sum_{r=1}^{N}w_{rj}$ is the sum of the connection weights between the input neurons *N* and the hidden neuron *j*, and *Q_ik_* represents the percentage of the influence of the input variable *x_i_* on the output neuron *y_k_* in relation to the other input variables so that the summation of this index must result in a value of 100% for all input data [48]. Furthermore, the set of connection weights and biases of the optimal ANN is given in Table 2.

**Figure 7:**
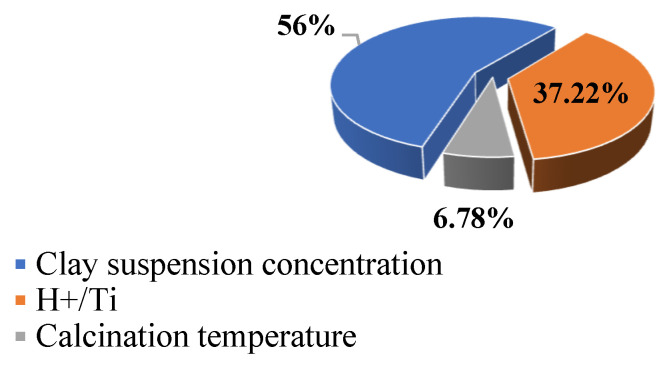
The relative contribution of each factor in determining the specific surface area of Ti-PILC.

Figure 7 shows that of all three parameters examined, the highest contribution percentage within the given ranges of change was 56% and related to the proportion of clay suspension. The maximum BET surface area was achieved with 0.5% (*w*/*v*) clay suspension; however, the clay suspension concentration of 3% (*w*/*v*) was also appropriate at a different H^+^/Ti ratio. Ti-PILC synthesis with diluted suspension leads to considerable porous properties on the one hand, and increases water consumption, production of wastewater, and complexity in separation and filtration on the other hand. It also requires more capacity and space for practical operations. Due to these economic concerns, working with more concentrated clay suspensions is important. Although the maximum BET surface area belongs to a sample prepared with 0.5% (*w*/*v*), other clay suspension concentrations 1, 3, and 10% (*w*/*v*) led to Ti-PILCs with S_BET_s of 135, 150 m^2^/g (both at H^+^/Ti = 4), and 106 m^2^/g (at H^+^/Ti = 2). As can be observed, another economically advantageous aspect of using concentrated clay suspension is the relatively low acid consumption. 

In addition to the percentage of clay suspension, the H^+^/Ti ratio with 37% contribution was the other effective parameter in the Ti-PILCs synthesis. The calcination temperature had the smallest influence on the system performance with only 7%. The effects of changing these parameters on Ti-PILCs have been discussed in detail in Section 4.1. The quantitative results obtained confirmed each parameter’s ratio, as shown in Figure 7.

### 4.3. Porosity Analysis

**Figure 8:**
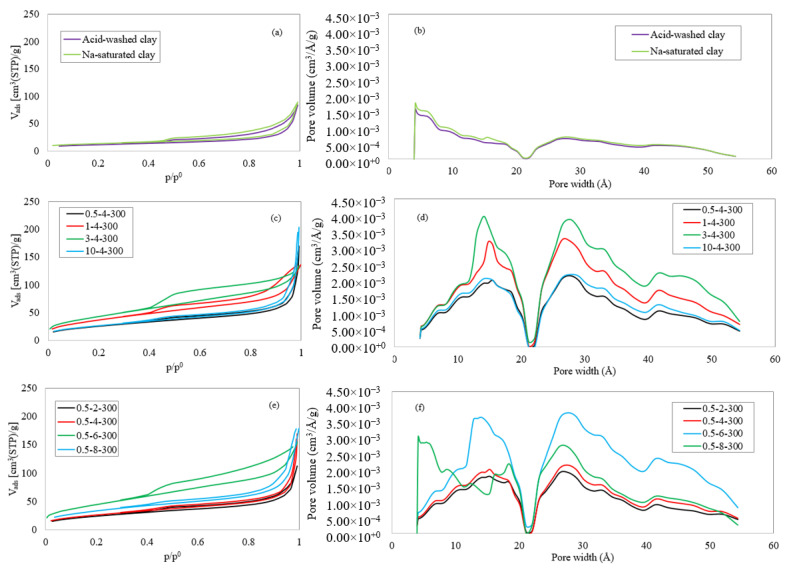
N_2_ adsorption–desorption isotherms of (**a**) acid-washed and Na-saturated bentonites, and Ti-PILCs produced under different (**c**) clay suspension concentrations, and (**e**) H^+^/Ti ratios; DFT pore size distribution curves of (**b**) acid-washed and Na-saturated bentonites, and Ti-PILCs produced under different (**d**) clay suspension concentrations, and (**f**) H^+^/Ti ratios.

Figure 8a shows the nitrogen adsorption–desorption isotherms of acid-washed raw bentonite and its Na-saturated form. According to the figure and the porous properties of Table 3, the raw clay has a mesoporous structure with an average pore diameter of about 130 Å. For these two samples, a relatively low specific BET surface area (approx. 40 m^2^/g) was obtained, which shows the minor role of the acid-washing and sodium-saturation steps in developing the porosity of clays. According to the slight initial slope of the isotherm curves (Figure 8a) and Table 3, the percentage of microporosity is extremely low in these samples. The hysteresis loops further confirmed the presence of mesopores at p/p^0^ greater than 0.4. Based on the IUPAC classification, the hysteresis loops are more similar to the H_3_ type than the others. It means that the mesopores in the clay have a slit shape [49].

A comparison of the adsorption–desorption isotherms of the titanium PILCs (Figure 8c,d) with the raw clay (Figure 8a) shows that the isotherms have a relatively similar shape, with an improvement in the N_2_ volume adsorbed at every region of p/p^0^. Depending on the operating conditions, the isotherm curves’ initial and final slopes and the hysteresis loops’ sizes are different. Investigation of the clay suspension concentration’s effect showed that the sample produced with the highest clay-suspension concentration has a greater total pore volume (0.32 cm^3^/g) related to its steeper curve at high pressures. An increase in clay suspension concentration (0.5 to 3% (*w*/*v*)) is associated with an increase in the percentage of micropores and narrowing of available pores. In contrast, the mesopore volume decreases, and the average mesopore size shifts to larger pores. Generally, 3-4-300 has a higher microporosity than the others, which could be the reason for the larger surface area. The initial part of the isotherm of this sample confirms the presence of a relatively large number of micropores in the porous network. Sample 10-4-300 has a mesoporous texture, with larger mesoporous and total pore volume. Changes in the H^+^/Ti ratio also lead directly to micro- and mesoporous volume variations. The sample 0.5-6-300 with the smallest average pore size (58.4 Å) and the highest micropore volume has the largest S_BET_.

Figure 8b,d,f shows that all samples’ DFT pore size distribution curves follow a uniform trend, with some differences in the percentages of the individual pore types. These curves divide the porous network into pores smaller than 22 Å (5–22 Å) and larger than (22–55 Å). In the case of nonpillared samples, the volume of both types is relatively small, but increases during pillarization. Almost all Ti-pillared clays have PSD curves with three local maxima, except 0.5-8-300, with an additional maximum at very small pore sizes.

**Figure 9:**
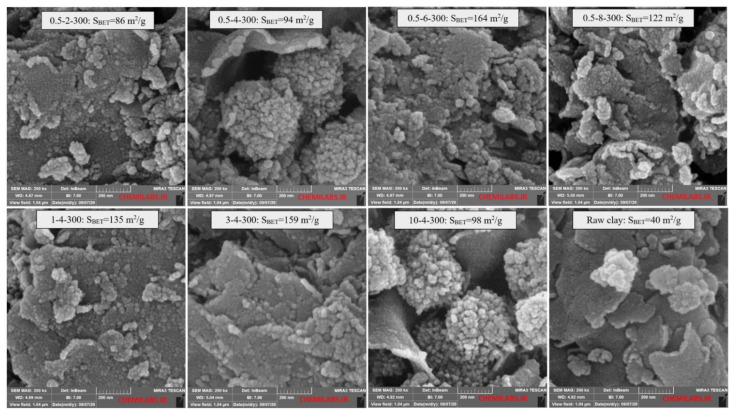
FESEM micrographs of natural and Ti-pillared clays prepared under different conditions.

**Figure 10:**
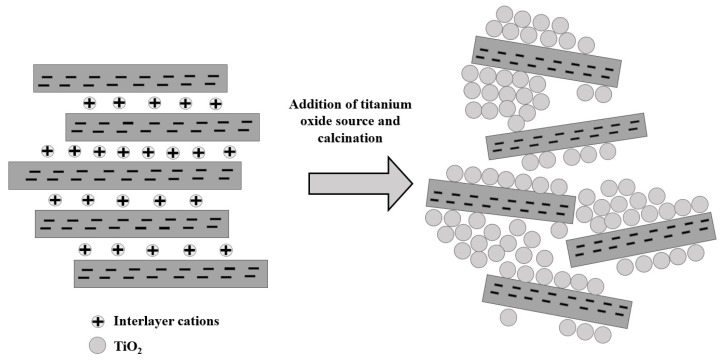
Different possibilities in the introduction of TiO_2_ particles into the raw clay layered texture.

FESEM micrographs of the raw clay compared to the Ti-PILCs produced under different operating conditions are shown in Figure 9. It can be observed that the raw clay with a smoother surface has a different structure than the Ti-PILCs. Comparing the raw clay with the Ti-PILC samples shows that the TiO_2_ particles are added to the clay surface as external nanoparticles, stuck together as a clog, and dispersed on the clay layers. Investigation of the FESEM micrographs shows that the Ti-PILC samples with compact clogs of TiO_2_ nanoparticles have a smaller specific surface area; however, in cases when these particles are dispersed as fine spots on the total surface of the samples, the specific surface area will increase. If it is associated with small mesopores and fractures, the higher surface areas can be obtained, for example, in 0.5-6-300, and 3-4-300. In contrast, the lack of these fractures or the presence of larger and more expanded pores can decrease the S_BET_ in the samples: 1-4-300 and 0.5-8-300. Figure 10 shows a schematic of different possibilities in the introduction of TiO_2_ and the change of internal structure of clay after intercalation of titanium source and calcination.

### 4.4. XRD and XRF Analysis

**Figure 11:**
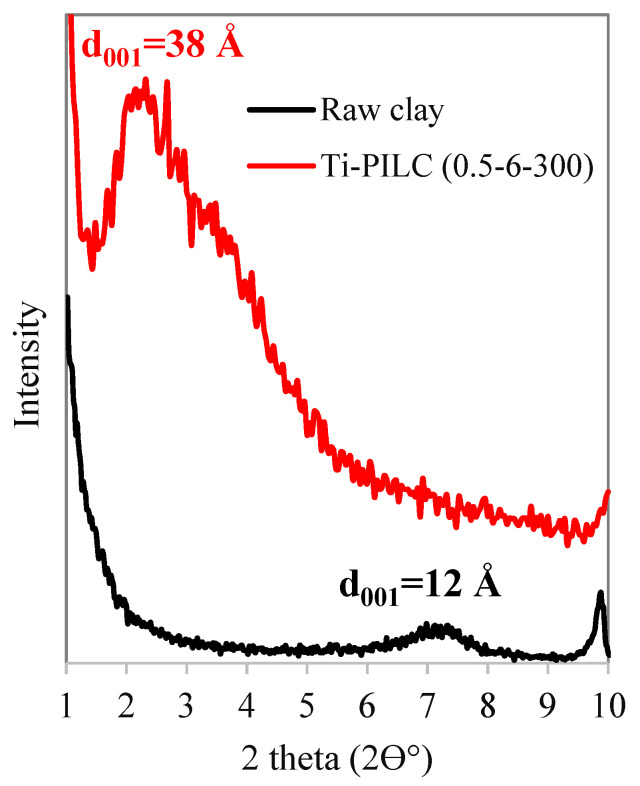
Low-angle X-ray diffraction and basal spacing of the raw clay and Ti-PILC (0.5-6-300).

In order to prove the role of TiO_2_ introduction as a pillaring agent, the low-angle XRD analysis at 2θ = 1–12° was performed on the raw clay, and Ti-PILC has the highest S_BET_ (0.5-6-300); the patterns are shown in Figure 11. It can be observed that the raw clay has an intense basal d_001_ reflection at 2θ~7°, with a basal distance of around 12 Å. For Ti-PILC, increasing the basal distance to around 38 Å was proven by the peak shifting to a much smaller 2θ (2–3°). Increasing the distance between layers and forming permanent interlayer distances are related to introducing TiO_2_ pillars to the raw clay structure. 

**Figure 12:**
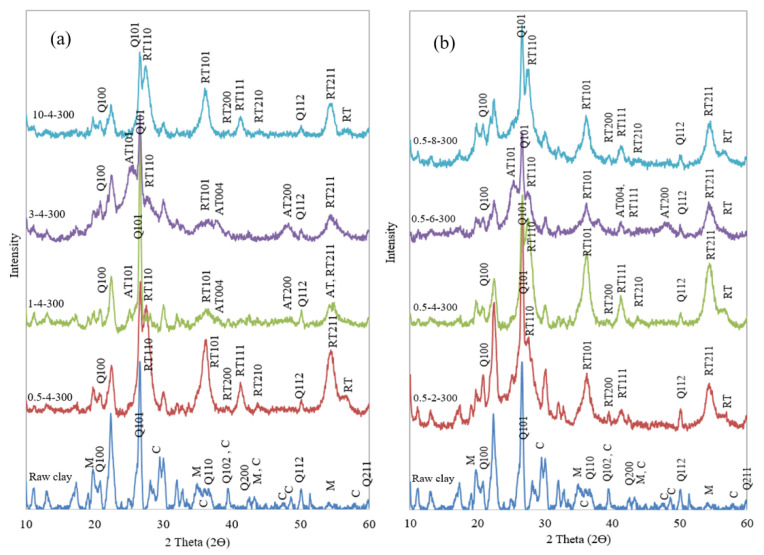
XRD patterns of Ti-pillared clays prepared under different operating conditions in comparison with the raw clay; effect of (**a**) clay suspension concentrations, and (**b**) H^+^/Ti ratio, AT: Anatase TiO_2_; RT: Rutile TiO_2_; Q: Quartz (SiO_2_); M: Montmorillonite; C: Calcite.

The normal XRD patterns of the Ti-PILC samples compared with the raw clay at large angles (2θ = 10–60°) are also shown in Figure 12. The presence of quartz (Q) and montmorillonite (M) was determined by detecting several peaks in the XRD pattern of the raw clay. It means that the pillaring process did not lead to the destruction of the main structure of the clay. On the other hand, the raw clay sample showed several peaks associated with the calcite (C) phase, which in the case of the Ti-PILCs, were eliminated or reduced in intensity due to the washing and pretreatment steps. Titanium in the form of TiO_2_ polymorphs was detected in the structure of all Ti-PILCs. Titanium oxide was found in all samples, mainly in the rutile phase, with significant peaks at 2θ = 27.6–27.8° (110), 35.8–36.4° (101), and 54.6° (211). In several samples, including 0.5-6-300, 1-4-300, and 3-4-300, additional peaks attributed to the anatase phase were also found at 2θ = 25.3° (110) and 48.8–48.9° (200) with relatively low intensity in addition to the rutile phase peaks. The more distinct presence of the rutile TiO_2_ phase is related to the heat treatment of the Ti-intercalated clays during the calcination step [50]. Since one of the critical properties of Ti-PILCs in addition to adsorption is the ability of metal sites to oxidize or photo-oxidize the adsorbed contaminants, the activity of titanium oxide sites should be investigated. However, both the anatase and rutile TiO_2_ phases are the active crystal phases of TiO_2_ [51]; concerning the higher (photo)catalytic activity of anatase TiO_2_ [52], Ti-PILC samples (such as 0.5-6-300), containing anatase in addition to rutile, are superior to the others. 

Figure 12a shows that the intensity of the rutile phases of TiO_2_ was very high for 0.5-4-300 and 10-4-300 (when the weight percentage of the clay suspension is at the lowest or highest value of the selected range), confirming the high percentage of TiO_2_ in the results of the XRF analysis. Figure 12b also compares the spectra for the samples prepared under different H^+^/Ti ratios. The highest intensity of the rutile phases was obtained for the pillared sample (0.5-4-300), containing about 52.2 wt.% titanium oxide (Table 4).

**Figure 13:**
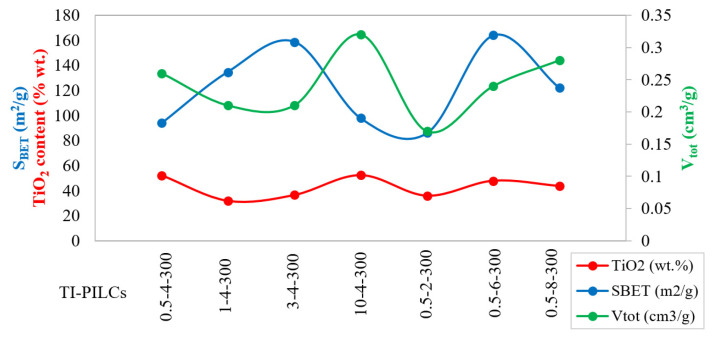
The relationship between the TiO_2_ content of Ti-PILCs and their porous properties.

The XRF analysis results (Table 4) showed the increase in the TiO_2_ content in the Ti-PILCs to about 31–52 wt.%. The highest TiO_2_ percentage was obtained for 0.5-4-300 and 10-4-300. The comparison of specific surface area and titanium oxide content showed a weak correlation between these two factors; in contrast, a stronger direct relationship between TiO_2_ content and total pore volume was observed (Figure 13). According to Table 4, a decrease in the weight percentages of silica, alumina, iron oxide, and calcite was observed during the pillaring process. The removal of calcite is one of the main objectives of the acetic acid washing step, which XRF data can confirm.

### 4.5. FTIR Analysis

**Figure 14:**
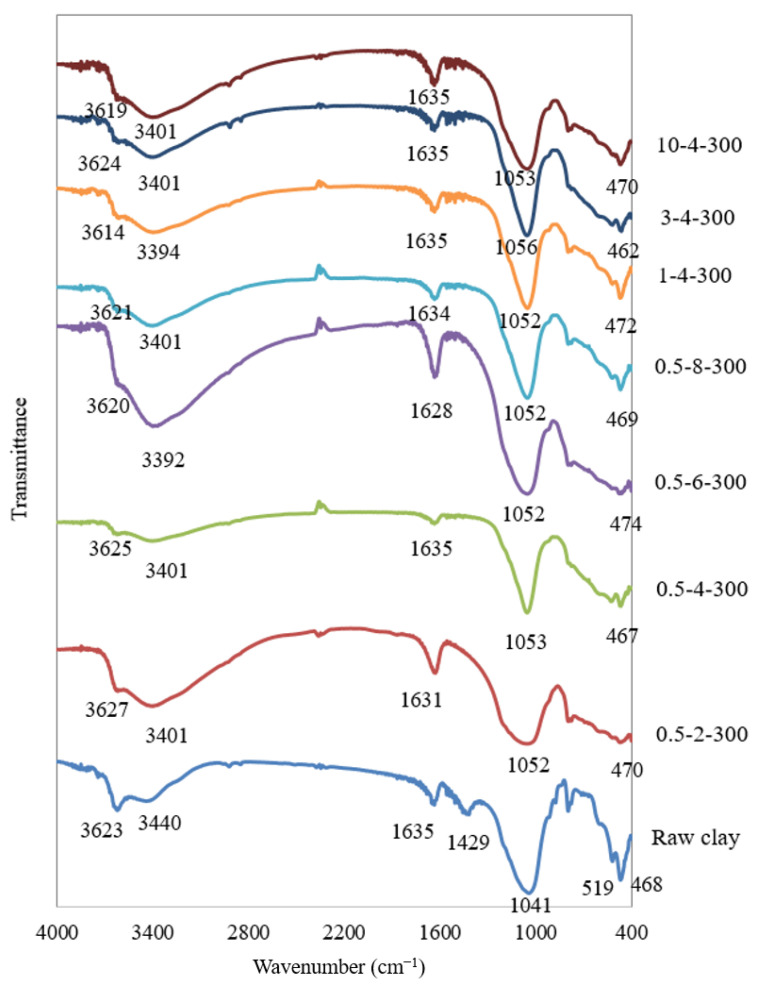
FTIR spectra of raw clay in comparison with the titanium-pillared clays.

The FTIR spectra (400–4000 cm^−1^) of the raw bentonite and some of the titanium pillared clays are shown in Figure 14. One of the main differences in the FTIR spectra of Ti-PILCs and raw bentonites is the bands occurring around 3600 and 3400 cm^−1^. The raw bentonite shows two peaks at 3623 and 3440 cm^−1^ due to the -OH stretching vibrations of the functional hydroxyl groups and the H_2_O-adsorbed molecules of the interlayer, respectively [26,36]. For the Ti-PILCs, however, these peaks shifted in all cases to lower wavenumbers (in the range of 3614–3627 cm^−1^ and 3392–3401 cm^−1^, respectively). This shift is accompanied by a decrease in the first band’s intensity (related to the -OH groups) and the second band’s widening (related to the H_2_O-adsorbed molecules). The Ti-pillarization, which is accompanied by the exchange of a large number of interlayer cations, usually in hydrated form, by poly(oxy-cations), is the reason for the change in the properties of the -OH-related bands. It also confirms dehydration and dehydroxylation of the raw clays during the pillarization process. Pillaring reduced the affinity of the clays to adsorb water and swelling. 

On the other hand, the bending vibrations of hydroxyl groups and interlayer water molecules in natural bentonite can be attributed to a relatively large band at about 1635 cm^−1^ [26]. In pillared clays, the corresponding peak between 1628–1635 cm^−1^ occurred with a relatively lower intensity. The raw clay spectra showed a band at 1400 cm^−1^ wavenumber, which was not observed in Ti-PILCs. It is attributed to bending vibrations of water removed after pillaring [53]. Si-O-Si is one of the main functional groups of clays; the related stretching vibration leads to the appearance of peaks in the spectra at about 1041 cm^−1^ for the raw clay and in the range of 1052–1056 cm^−1^ for the pillared clays. Si-O bending vibrations also occur at 468 cm^−1^ for the raw clay and are shifted to lower wavenumbers (462–474 cm^−1^) for Ti-PILCs. The band appeared at 519 cm^−1^ for the raw clay, attributed to the Al-O stretching vibrations [26], and was transferred into the range of 519–528 cm^−1^ after pillarization. In most cases, the intensity of these bands decreased significantly. 

## 5. Conclusions

Titanium-pillared clays were prepared and characterized to optimize the operating parameters such as clay suspension weight percentage, acid/Ti molar ration, and calcination temperature. A local Iranian clay and titanium isopropoxide were applied at relatively high concentrations of clay suspension to achieve suitable conditions for a more sustainable process with lower water and energy consumption. Although the highest BET surface area (164 m^2^/g) was obtained for the Ti-PILC sample prepared with the most dilute clay suspension (0.5% (*w*/*v*)), achieving a reasonable S_BET_ (159 m^2^/g) was also possible at a higher concentration of clay suspensions (3% (*w*/*v*)). At this condition, a relatively low H^+^/Ti ratio was sufficient to obtain a sufficiently large surface area, which is another advantage. Pillarization with titanium isopropoxide under the conditions proposed in the present work increased the SBET of the raw clay to about 273%. It also increased the basal spacing from 12 Å to 38 Å due to the insertion of TiO_2_ nanoparticles as pillars. The sensitivity analysis showed that the calcination temperature in the range of 300 to 700 °C had a negligible negative effect on the porous properties of the Ti-PILC sample, and 300 °C gave the best S_BET_. The titanium oxide content in the structure of Ti-PILCs with the best porous properties was about 31–52 wt%, containing both rutile and anatase phases. These samples exhibited a mesoporous structure with an average pore size of about 55 ± 3 Å, an isotherm of type (IV), and a hysteresis loop (type H_3_). The production process was successfully modeled using ANN and the Levenberg–Marquardt algorithm (LMA) with seven hidden layer neurons. The most influential factor in the performance of the titanium pillarization process was the clay suspension, followed by the ratio of H^+^/Ti.

## Figures and Tables

**Table 1:** Comparison of the highest specific surface area obtained in the present study with other Ti-PILCs from the literature.

Titanium Source *	Raw Clay S_BET_ (m^2^/g)	Ti-PILC S_BET_ (m^2^/g)	Ti-PILC V_tot_ (cm^3^/g)	S_BET_ Enhancement after Pillaring (%)	Ref.
TC	70	185–360	-	414	[24]
TIP	152	298–404	-	166	[38]
TC	91	280	-	208	[39]
TIP	85	250–283	0.18–0.23	231	[40]
TIP	90	151	-	68	[41]
TC	77	212	0.22	175	[42]
TB	10	136	0.16	1235	[43]
TE	117	208	-	78	[21]
TIP	77	149	0.14	92	[22]
TIP	57	191–373	0.22–0.32	557	[26]
TE	74	230–334	-	211–351	[27]
TNP		190–306	-	157–313	
TIP		146–273	-	97–269	
TB		124–265	-	68–258	
TIP	104	105	0.13	1	[30]
TIP	23	66	0.10	187	
TB	149	157	0.23	5	[44]
TC	49	329	0.14	571	[45]
TC	65	216	-	232	[5]
TIP	44	164	0.13–0.45	273	This paper

* Titanium tetrachloride (TC); Titanium tetraisopropoxide (TIP); Titanium normal propoxide (TNP); Titanium butoxide (TB); Titanium ethoxide (TE).

**Table 2:** Weight matrix of the designed ANN.

Number of Neurons in the Hidden Layer	Weights and Biases between Input and Hidden Layers				Weights and Biases between Hidden and Output Layers	
	Normalized Input Variables			Bias	Normalized Output S_BET_	Bias
	Clay Suspension Concentration	H^+^/Ti	Calcination Temperature			
1	1.6329	−2.1759	0.1419	−3.1969	1.7711	1.0061
2	−2.4581	0.0579	−0.4061	0.4805	3.1642	
3	0.9654	1.2839	−0.0339	0.9716	5.9584	
4	−6.8028	−2.7467	0.1051	−4.8613	2.2528	
5	−0.5566	2.5503	−0.9633	0.8387	0.0390	
6	−2.3382	2.6971	−0.0558	−1.5031	−3.1359	
7	−5.7059	0.1926	−0.1625	−7.0700	3.3928	

**Table 3:** Porous properties of Ti-PILCs obtained at different operating conditions in comparison with the acid-washed and Na-saturated bentonites *.

Sample		SBET (m^2^/g)	Vtot (cm^3^/g)	Vmic (cm^3^/g)	Vmes (cm^3^/g)	dmic. (Å)	dmes. (Å)	davg. (Å)
Acid-washed Bentonite		40	0.13	0.02	0.13	13.8	36.7	130.3
Na-saturated Bentonite		44	0.14	0.02	0.13	12.6	37.2	125.4
Ti-PILC	0.5-2-300 **	86	0.17	0.03	0.16	12.6	34.2	80.9
	0.5-4-300	94	0.26	0.04	0.24	12.6	34.0	112.2
	0.5-6-300	164	0.24	0.06	0.22	10.0	34.3	58.4
	0.5-8-300	122	0.28	0.05	0.25	12.9	34.0	91.0
	1-4-300	135	0.21	0.05	0.19	11.6	34.3	62.0
	3-4-300	159	0.21	0.06	0.19	10.3	34.4	52.5
	10-4-300	98	0.32	0.04	0.30	12.7	34.1	129.3

* S_BET_: BET specific surface area; V_tot_ = Total pore volume (p/p^0^ > 0.99); V_mic_ = Micropore volume; Micropores (%): percentage of microporosity; d_mic_ = Average micropore size; d_mes_ = Average mesopore size; d_avg_ = Average pore size. ** Clay suspension concentration (% *w*/*v*)-H^+^/Ti ratio-Calcination temperature.

**Table 4:** Chemical composition of natural bentonite and Ti-Pillared bentonite prepared under different operating conditions.

Sample	Weight Percentage (%)							
	SiO_2_	Al_2_O_3_	Fe_2_O_3_	CaO	Na_2_O	K_2_O	MgO	TiO_2_
Raw Clay	74.6	11.7	2.3	5.7	1.8	0.9	2.1	0.3
0.5-4-300	38.5	5.7	1.2	0.5	0.1	0.3	1.1	52.2
1-4-300	55.5	8.5	1.2	0.9	0.2	0.5	0.9	31.9
3-4-300	50.1	9.6	1.3	0.6	0.2	0.4	1.1	36.5
10-4-300	38.4	5.7	1.1	0.4	0.2	0.4	1.1	52.3
0.5-2-300	52.3	7.1	1.2	0.9	0.3	0.7	1.0	36.0
0.5-6-300	41.5	6.8	1.2	0.6	0.2	0.3	1.1	47.9
0.5-8-300	45.2	6.8	1.4	0.7	0.2	0.3	1.3	43.7
